# Thermal plasticity of the miRNA transcriptome during Senegalese sole development

**DOI:** 10.1186/1471-2164-15-525

**Published:** 2014-06-25

**Authors:** Catarina Campos, Arvind YM Sundaram, Luisa MP Valente, Luis EC Conceição, Sofia Engrola, Jorge MO Fernandes

**Affiliations:** CIIMAR/CIMAR, Centro Interdisciplinar de Investigação Marinha e Ambiental and ICBAS–Instituto de Ciências Biomédicas de Abel Salazar, Rua dos Bragas, Porto 289, 4050-123 Portugal; Faculty of Biosciences and Aquaculture, University of Nordland, Bodø, 8049 Norway; CCMAR/CIMAR, Centro de Ciências do Mar, Faro, 8005-139 Portugal; Sparos Lda, CRIA, Universidade do Algarve, Campus de Gambelas, Faro, 8005-139 Portugal

**Keywords:** Solea senegalensis, miRNA, Embryonic temperature, Myogenesis, Growth, Epigenetics

## Abstract

**Background:**

Several miRNAs are known to control myogenesis in vertebrates. Some of them are specifically expressed in muscle while others have a broader tissue expression but are still involved in establishing the muscle phenotype. In teleosts, water temperature markedly affects embryonic development and larval growth. It has been previously shown that higher embryonic temperatures promoted faster development and increased size of Senegalese sole (*Solea senegalensis*) larvae relatively to a lower temperature. The role of miRNAs in thermal-plasticity of growth is hitherto unknown. Hence, we have used high-throughput SOLiD sequencing to determine potential changes in the miRNA transcriptome in Senegalese sole embryos that were incubated at 15°C or 21°C until hatching and then reared at a common temperature of 21°C.

**Results:**

We have identified 320 conserved miRNAs in Senegalese sole, of which 48 had not been previously described in teleosts. mir-17a-5p, mir-26a, mir-130c, mir-206-3p, mir-181a-5p, mir-181a-3p and mir-199a-5p expression levels were further validated by RT- qPCR. The majority of miRNAs were dynamically expressed during early development, with peaks of expression at pre-metamorphosis or metamorphosis. Also, a higher incubation temperature (21°C) was associated with expression of some miRNAs positively related with growth (e.g., miR-17a, miR-181-5p and miR-206) during segmentation and at hatching. Target prediction revealed that these miRNAs may regulate myogenesis through MAPK and mTOR pathways. Expression of miRNAs involved in lipid metabolism and energy production (e.g., miR-122) also differed between temperatures. A miRNA that can potentially target *calpain* (miR-181-3p), and therefore negatively regulate myogenesis, was preferentially expressed during segmentation at 15°C compared to 21°C.

**Conclusions:**

Temperature has a strong influence on expression of miRNAs during embryonic and larval development in fish. Higher expression levels of miR-17a, miR-181-5p and miR-206-3p and down-regulation of miR-181a-3p at 21°C may promote myogenesis and are in agreement with previous studies in Senegalese sole, which reported enhanced growth at higher embryonic temperatures compared to 15°C. Moreover, miRNAs involved in lipid metabolism and energy production may also contribute to increased larval growth at 21°C compared to 15°C. Taken together, our data indicate that miRNAs may play a role in temperature-induced phenotypic plasticity of growth in teleosts.

**Electronic supplementary material:**

The online version of this article (doi:10.1186/1471-2164-15-525) contains supplementary material, which is available to authorized users.

## Background

MicroRNAs (miRNAs) are short non-coding regulatory RNAs that regulate gene expression post-transcriptionally. Mature miRNAs are often conserved across a wide range of species, and mutations in proteins required for miRNA function or biogenesis have shown to impair animal development [1]. They are involved in the majority of physiological processes, including stem cell differentiation, cell lineage specification, haematopoiesis, neurogenesis, immune response and myogenesis [1]. MiRNA-mediated gene regulation involves binding to the 3’-UTR of their mRNA target and sequestration of mRNA by the miRNA/RISC complex (RNA-induced silencing complex**)** in the cytoplasmic processing bodies, which results in repression of translation initiation or mRNA degradation reviewed in [2].

In vertebrates, somatic and skeletal muscle growth is strongly stimulated by Igf-I (Insulin-like growth factor I) [3], whose functions are mediated by the Igf-I receptor (Igf1R) through activation of two major intracellular signalling pathways: mitogen-activated protein kinases (MAPKs) and mTOR (mammalian target of rapamycin) through phosphatidylinositol 3 kinase (PI3K)/Akt [4]. mTOR mediates signalling in response to nutrient availability, cellular energy, mitogenic signals and various types of stressors. Phosphorylation of mTOR increases levels of protein synthesis by regulating essential proteins controlling mRNA translation. The MAPK pathway is essential for muscle cell proliferation and differentiation [5, 6], and the closely related hepatic insulin pathway acts upstream of Akt/mTOR. It recruits IRS proteins, which in turn recruit PI3K to phosphorylate Akt. Downstream of Akt, subsequent metabolic effects are largely mediated by mTOR and also FoxO1 [7]. mTOR then regulates S6K1 and 4-EBP1 to induce hepatic protein synthesis and to stimulate lipogenesis [7].

The physiological actions of these complex pathways are multiple and several miRNAs have already been identified as intervenients on their regulation. For instance, in human, miR-199a-3p and miR-101 can suppress gene expression by directly binding to the 3’-UTR of mTOR [8, 9]. Furthermore, miR-181 regulates PTEN, the principal negative regulator of PI3K [10]. MiRNAs that influence mTOR and MAPK pathways play crucial roles in myogenesis, since they are essential for muscle development and growth. For example, miR-181 is involved in myoblast differentiation and in establishing the muscle phenotype [11]. Additionally, some miRNAs (e.g., miR-1, miR-133 and miR-206) are specifically expressed in muscle and positively related with its growth [12]. Interestingly, in teleost like zebrafish (*Danio rerio*), fast muscle miRNA populations are differentially regulated during the transition from hyperplastic to hypertrophic muscle phenotype in adult fish [13]. Also, the dynamic expression of miR-206, miR-26a and miR-214 throughout skeletal muscle development in the common carp (*Cyprinus carpio*), points to an active role of these miRNAs in the myogenic process [14].

miRNAs can also modulate protein abundance and/or phosphorylation status of key signaling components of the insulin pathway. Expression of miR-122 [15] and miR-33 [16] is inversely correlated with expression and/or phosphorylation status of the α-subunit of the metabolic sensor AMPK. In fact, miR-122, which is highly abundant in mammalian [17] and fish liver [18], exerts a stimulatory role on lipogenesis and cholesterol synthesis, and inhibits fatty-acid oxidation and therefore energy production [15].

In fish, changes in miRNA expression have been documented during early ontogeny [19, 20] or in response to food ingestion [18]. However, the potential effect of water temperature on the miRNA transcriptome has never been investigated in teleosts, in spite of its profound impact on larval and juvenile growth [21]. The flatfish Senegalese sole (*Solea senegalensis*) is a species of commercial interest for marine aquaculture, particularly in Southern Europe, and it can be exposed to large thermal variations in natural and aquaculture conditions [22]. In a previous study it was shown that higher embryonic temperatures (18 and 21°C) promoted a faster development and increased the size of sole larvae by 30 days post hatch (dph) relatively to a lower temperature (15°C) [23]. Furthermore, muscle cellularity at equivalent developmental stages was affected by temperature and a transient differential gene expression associated with incubation temperature was also observed at several stages. For example, *myf5*, *fst*, *myHC* and *mrf4* transcript levels were higher at 21°C compared to 15°C [23]. It is likely that this differential expression of myogenic genes is regulated by miRNAs to some extent but their role in thermal plasticity of development and growth remains to be investigated. Hence, we have used the SOLiD deep-sequencing platform to determine potential changes in the miRNA transcriptome in Senegalese sole embryos and larvae exposed to two different temperatures (15°C or 21°C) during embryonic development. Moreover, we have predicted targets of miRNAs that were differentially expressed with temperature, in order to understand their potential role in the phenotypic plasticity of growth in teleosts.

## Results

### SOLiD sequencing of small RNAs

**Figure 1:**
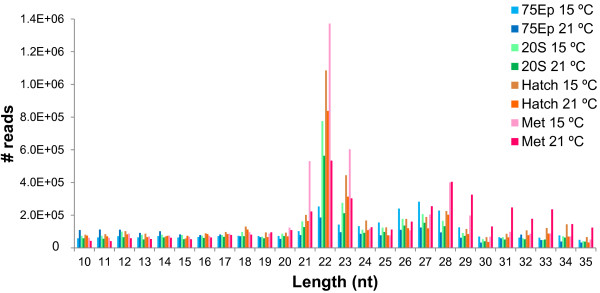
**Histogram of read lengths (nt, nucleotides) obtained by SOLiD sequencing of Senegalese sole RNA samples.** After trimming, two peaks in read length were found for all samples: one at 22–23 nt and a smaller one at 28–29 nt, which correspond to miRNAs/short interfering miRNAs and piwi-interacting RNAs, respectively. The majority of reads were around the 22 nt peak, indicating miRNA enriched samples. Embryos were incubated at 15°C or 21°C and transferred to 21°C after hatching. The developmental stages analysed were 75% epiboly (75Ep), 20 somites (20S), hatching (hatch) and metamorphosis (met).

To identify Senegalese sole miRNAs expressed during development, 8 small RNA libraries were prepared and sequenced using the SOLiD deep-sequencing platform. A total of 55,053,966 raw reads were obtained, resulting in 27,639,077 reads after sequence trimming. All small RNA-seq data have been deposited in Gene Expression Omnibus under the accession GSE58297. After discarding rRNAs, tRNAs and snoRNAs, 320 conserved miRNAs from 149 miRNA families were identified (Table 1). It is noteworthy that 48 of these miRNAs had not been previously described in fish (Additional file 1: Table S1). Read length distributions for each sample are shown in Figure 1. Two peaks in read length were found for all samples, one at 22–23 nt and another around 28–29 nt, which correspond to miRNAs/interfering miRNAs and piwi-interacting RNAs, respectively. The majority of reads were around the 22 nt peak, indicating miRNA enriched samples (Figure 1). No novel miRNAs were identified amongst the publicly available nucleotide sequences from Senegalese sole.

**Table 1:** **Summary of the sequenced miRNA transcriptomes in Senegalese sole embryos and larvae incubated at 15°C or 21°C**

Sample	# Total reads	# Trimmed (% Total)	Average length (nt)	# Annotated miRBase (% Total)	# Conserved miRNAs
**75Ep 15°C**	6 642 089	2 802 986 (42.2)	23.5	200 224 (3.0)	232
**75Ep 21°C**	8 842 550	2 169 069 (24.5)	21.1	174 027 (2.0)	231
**20S 15°C**	6 566 636	3 320 266 (50.6)	22.4	993 981 (15.1)	265
**20S 21°C**	6 101 835	2 564 327 (42.0)	22.7	689 954 (11.3)	265
**Hatch 15°C**	7 268 950	4 312 425 (59.3)	23.0	1 467 039 (20.2)	285
**Hatch 21°C**	6 200 277	3 256 433 (52.5)	22.4	1 162 239 (18.7)	281
**Met 15°C**	7 105 697	4 965 849 (69.9)	23.0	2 391 674 (33.7)	288
**Met 21°C**	6 325 932	4 247 722 (67.2)	25.2	804 587 (12.7)	279

75Ep: 75% epiboly, 20S: 20 somites, Hatch: hatching, Met: metamorphosis stage 3.

### Differential expression of conserved miRNAs during Senegalese sole development and with embryonic temperature

**Figure 2:**
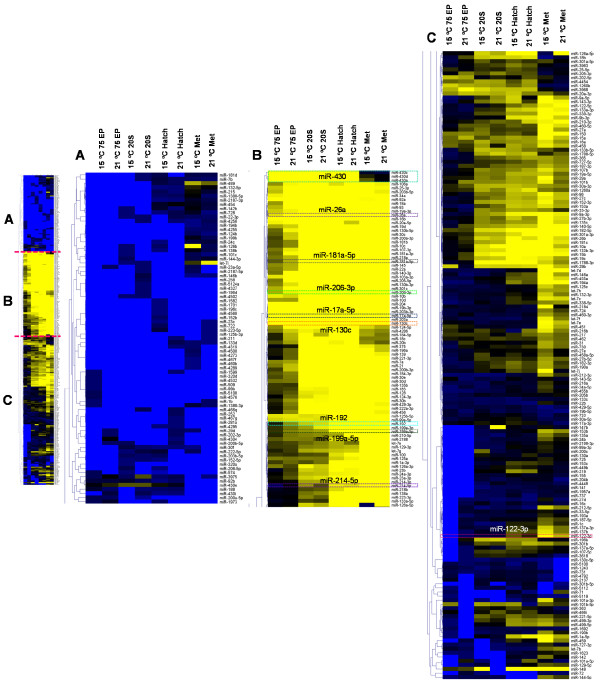
**Heatmap of conserved miRNAs obtained by SOLiD sequencing during Senegalese sole early development.** The complete heatmap (normalised to the number of trimmed reads, log2 transformed, Euclidean distance, complete linkage) has 320 rows (miRNAs) and 8 columns that correspond to 4 developmental stages (75Ep, 20S, hatching and metamorphosis) and two embryonic temperatures (15 and 21°C). For visualisation purposes, the heatmap was split in 3 panels: A) miRNAs with a low number of reads throughout development, B) miRNAs consistently expressed at high levels at all stages, and C) miRNAs with variable expression through development. Yellow and bue indicate higher and lower expression levels, respectively. miRNAs frequently referred to in the main text are highlighted in larger font.

**Figure 3:**
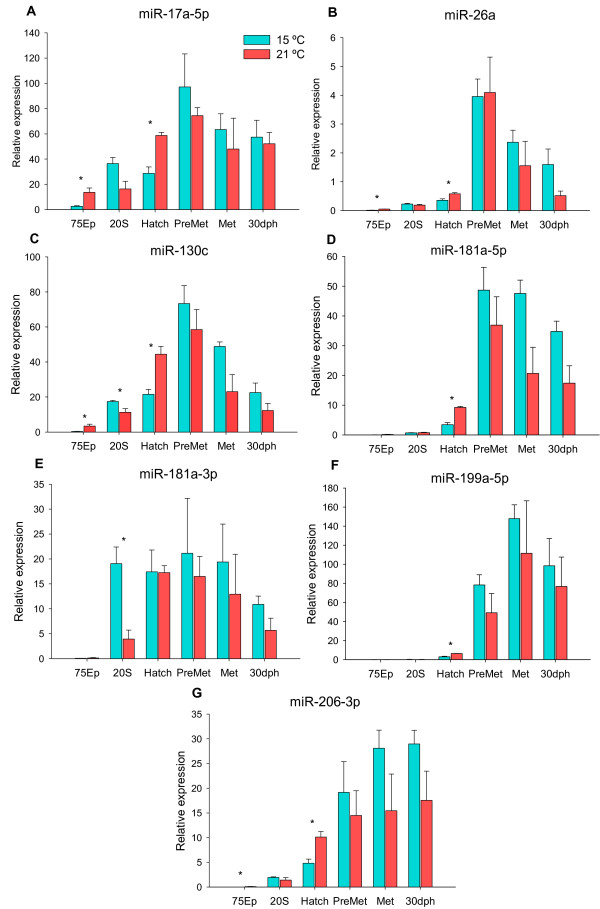
**Relative expressions of (A) miR-17a-5p, (B) miR-26a, (C) miR-130c, (D) miR-181a-5p, (E) miR-181a-3p, (F) miR-199a-5p and (G) miR-206-3p during Senegalese sole early development.** Embryos were incubated at 15°C (blue bars) or 21°C (red bars) and transferred to 21°C after hatching. Six developmental stages were analysed (75% epiboly (75Ep), 20 somites (20S), hatching (hatch), pre-metamorphosis (pre-met), metamorphosis (met) and 30 days post-hatch (30 dph)) (*N* = 3 pools of embryos or larvae). Transcript levels were determined by qPCR and the control RNA Spike-in (Exiqon) was used to compare miRNA expression profiles. Significant differences between temperatures are indicated by asterisks (*P* < 0.05).

Hierarchical clustering of the 320 conserved miRNAs found with SOLiD sequencing in Senegalese sole revealed three main clusters: miRNAs with very low number of reads throughout development (Figure 2A), miRNAs generally highly expressed in all developmental stages (Figure 2B) and miRNAs with variable expression during development (Figure 2C).miRNA diversity and expression varied amongst developmental stages (Figures 2, 3). Notably, several passenger strands showed very high expression values in one or more stages of development, such as miR-122-5p, miR-140-3p, miR-181a-3p, miR-199a-3p or miR-203b-5p (Figure 2B, C and Figure 3E). Generally, at earlier stages of development (i.e., 75Ep and 20S), there was less variety and lower level of miRNA transcripts than at later stages (Figure 2). The miRNA with highest number of reads during 75Ep in both temperature groups was miR-430d (3.1 and 1.6% of the total number of reads at 15 and 21°C, respectively) (Figure 2B), followed by miR-430a and 430c. The miR-430 family was prominently expressed until hatching, but its expression decreased sharply after that and at metamorphosis hardly any expression could be detected (Figure 2B). At 75Ep, the passenger strands with the highest number of reads were miR-203b-5p, miR-202-5p and miR-20a-3p (Figure 2B, C). During the 20S stage, miRNAs such as miR-203a, miR-17a-5p or miR-130c were found to be highly expressed (Figure 2B; Figure 3A, C). At hatching, miR-130c was the miRNA with the highest number of reads in both temperatures (2.4 and 6.3% of total miRNAs at 15 and 21°C, respectively). At this point, miR-203a, miR-204, miR-301c and miR-10d were also found in a large number of reads (Figure 2B). Other miRNAs were also mostly observed after hatching, such as miR-125, miR-199, miR-192, miR-214 and the let-7 family. During metamorphosis, miR-192 was the miRNA with the highest number of reads in the 15°C group (4.0%) and the second highest in the 21°C group (1.2%). miR-130c, miR-125b-5p, miR-199a-5p and miR-181a-5p were also amongst the miRNAs with highest expression at this stage (Figure 2B, Figure 3C, D, F). Likewise, the passenger strands miR-122-5p, miR-199a-3p, miR-140-3p and miR-181a-3p were expressed at high levels during metamorphosis (Figure 2B, Figure 3E). Furthermore, expression of some passenger and guide strands of the same miRNA differed during development. For example, miR-122-5p and miR-140-3p were generally expressed at higher levels throughout development than their guide strands, miR-122-3p and miR-140-5p, respectively (Figure 2B andC).The digital expression profiles obtained with SOLiD sequencing in Senegalese sole were validated by qPCR of selected miRNAs (r = 0.69, 0.76, 0.98, 0.91, 0.97, 0.98 and 0.81 for miR-17a-5p, mir-26a, miR-130c, miR-206-3p, miR-181a-5p, miR-181a-3p and miR-199a-5p, respectively). These miRNAs were selected based on: 1) previous knowledge of their functions in other species, namely involvement in development and growth, muscle differentiation or response to stress, and 2) differential expression during development and/or temperature, as evidenced by the above SOLiD sequencing results. Quantification of selected miRNAs was performed not only in the same samples used for SOLiD sequencing but also in pre-metamorphic and 30 dph larvae (Figure 3A-G). Most miRNAs analysed had the highest expression levels at the pre-metamorphosis stage (miR-26a, miR-17a-5p, miR-130c, miR-181a-5p and miR-181a-3p), whilst miR-199-5p and miR-206-3p were preferentially expressed during metamorphosis (Figure 3).

There were several significant differences in miRNA expression between embryonic temperatures. miR-17a-5p, miR-26a, miR-130c and miR-206-3p transcripts were significantly higher at 21°C than at 15°C during 75Ep (*P* < 0.05) (Figure 3A, B, C, G). At the 20S stage, miR-130c and miR-181a-3p showed a 1.5- and 4.9-fold higher expression at 15°C than 21°C (*P* < 0.05), respectively (Figure 3C, E). At hatching, miR-17a-5p, miR-26a, miR-130c, miR-181a-5p, miR-199a-5p and miR-206-3p were highly expressed at 21°C compared to 15°C (Figure 3A, B, C, D, F, G; *P* < 0.05). No significantly differences between temperatures were found after hatching.

### miRNA target prediction in Senegalese sole

**Figure 4:**
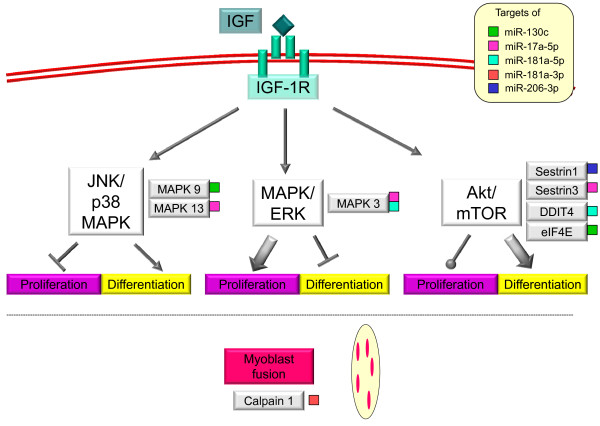
**Simplified model of predicted miRNA targets related to Calpain1, JNK/p38 MAPK, MAPK/ERK and Akt/mTOR pathways.** Igf functions are mediated by the Igf-I receptor (Igf1R) through activation of two major intracellular signalling pathways: MAPK and mTOR. mTOR and p38 MAPK are required for myogenic differentiation and p38 MAPK also inhibits proliferation. miR-130c was predicted to regulate *EIF4E* and *MAPK9*, miR-17a-5p may bind to the 3’UTRs of *Sestrin3*, *MAPK3* and *MAPK13*, miR-181a-5p could target *DDIT4* and *MAPK3*, miR-181a-3p may regulate *Calpain1* and miR-206-3p was predicted to target *Sestrin1*. In Senegalese sole, miR-17a, miR-181-5p and miR-206 levels were higher at 21°C than 15°C during somitogenesis, whereas miR-181a-3p was down-regulated at 21°C compared to 15°C. Arrows, circles and horizontal lines indicate activation, negligible effect and inhibition, respectively.

Several miRNAs had possible mRNAs targets belonging to or directly affecting MAPK and mTOR pathways (Figure 4, Table 2). In particular, miR-130c was predicted to regulate *eif4e* (eukaryotic translation initiation factor 4E) and *mapk9*, miR-17a-5p may bind to the 3’UTRs of *sestrin3*, *mapk3* and *mapk13*, miR-181a-5p could target *ddit4* (DNA-damage-inducible transcript 4) and *mapk3*, miR-181a-3p may regulate *calpain1* and miR-206-3p was predicted to target *sestrin1*.

**Table 2:** **Predicted targets of five differentially expressed miRNAs during early development of Senegalese sole**

miRNA	Ensembl Gene ID	Gene name	miRanda		RNAhybrid
			Score	Energy (kcal/mol)	Energy (kcal/mol)
miR-130c	ENSGACG00000005536	*EIF4E*	156	-25.65	-28.7
miR-130c	ENSGACG00000018076	*MAPK9*	142	-21.42	-24.4
miR-17a-5p	ENSGACG00000017027	*Sestrin3*	143	-24.03	-26.8
miR-17a-5p	ENSGACG00000013102	*MAPK3*	154	-21.58	-26.2
miR-17a-5p	ENSGACG00000004911	*MAPK13*	140	-22.15	-24.1
miR-181a-5p	ENSGACG00000019700	*DDIT4*	146	-20.26	-22.5
miR-181a-5p	ENSGACG00000013102	*MAPK3*	161	-20.51	-26
miR-181a-3p	ENSGACG00000018991	*Calpain 1*	156	-20.35	-31
miR-206-3p	ENSGACG00000008895	*Sestrin1*	144	-20.56	-22.7

Targets were predicted using miRanda and RNAhybrid. MiRanda score and minimum free energy (ΔG kcal · mol^-1^) calculated using miRanda and RNAhybrid are marked for each miRNA-3’-UTR target prediction.

## Discussion

### Characterisation of Senegalese sole miRNAs

Using high-throughput SOLiD sequencing, 320 conserved miRNA guide and passenger strands were identified and their expression profiles analysed in Senegalese sole embryos and larvae subjected to two different incubation temperatures (15°C and 21°C). There was some tendency to observe a higher number of conserved miRBase reads at later developmental stages and at a lower water temperature. This is unlikely to be a technical bias, since all libraries were prepared simultaneously and run on the same slide. Instead, it reflects a dynamic miRNA transcriptome throughout early ontogeny, which is influenced by environmental factors. From the 320 conserved miRNAs, 48 were not previously found in fish but were known in mammals, birds or nematodes. Up- or down-regulation of some of them is known to be involved in cancer-associated gene deregulation, namely miR-211 [24] and miR-509 [25]. miR-71 mediates the effects of germ cell loss on life span, and its over-expression was found to extend the life span of *Caenorhabditis elegans* lacking germ cells [26]. Most of these novel miRNAs were found in more than one small RNA library and were regulated, which indicates that they may play a key role in Senegalese sole development. Nevertheless, the specific functions of these miRNAs in embryogenesis remain to be ascertained.

There is increasing evidence that miRNAs that possess both highly conserved -5p and -3p sequences, especially within seed and anchor sequences, can originate two mature functional miRNAs, such as the case of miR-18a, miR-140 or miR-17 [27]. In the present study, several passenger strands were expressed at higher levels than the respective guide strands at one or more developmental stages (Figure 2 and 3), which suggests some involvement of these strands in Senegalese sole development.

### Expression profile and thermal plasticity of conserved miRNAs during Senegalese sole development

Critical early ontogeny events like organogenesis, hatching or metamorphosis in fish involve dramatic changes in signalling, physiology and morphology [28–31]. Well defined expression windows of myogenic and growth-related genes during Senegalese sole ontogeny and larval development have been previously characterised, since different genes are activated or down-regulated at different stages of development [23, 32]. It is plausible that allometric differences in growth of specific tissues may influence the observed miRNA expression profiles. Nevertheless, substantial changes in the Senegalese sole miRNA transcriptome during development (Table 1) highlight the importance of miRNAs in tissue and organ differentiation. miRNA differential expression has been found during the development of other vertebrate species, and similarly to our work, generally an increasing number of miRNAs are observed from the earliest to the latest stages of development [19, 33]. Furthermore, we found that whilst some Senegalese sole miRNAs were expressed throughout early ontogeny, others were stage-specific. The miR-430 family is an example of the latter (highly expressed only in earlier stages) and it is well known to accelerate deadenylation and clearance of maternal transcripts during zygotic stages [34, 35]. miRNAs mostly observed after hatching or during metamorphosis included miR-125, miR-199, miR-192, miR-214 and the let-7 family (Figure 2). The latter comprises a family of miRNAs that are highly conserved from worms to humans [36] and are involved in various processes, such as regulating cell proliferation and differentiation during development.

Several miRNAs showed an expression peak during pre-metamorphosis or metamorphosis (Figures 2 and 3). For instance, amongst the miRNAs validated by qPCR, miR-17a-5p, miR-26a, miR-130c,miR-181a-5p and miR-181a-3p showed a peak of expression mainly during the pre-metamorphic stage (Figure 3A, B, C, D, E), whereas, miR-199a-5p and miR-206-3p had higher levels at metamorphosis or later (Figure 3F, G). Similar results were found for some miRNAs in the olive flounder (*Paralichthys olivaceus*) [37]. Flatfish larvae undergo a particularly impressive metamorphosis, resulting in an asymmetrical craniofacial remodelling and lateralized behaviour [38, 39]. In olive flounder, an involvement of miR-206a has also been suggested in the metamorphic process, which is mediated by the thyroid hormone [40]. It is known that Senegalese sole larvae have a high growth rate and accumulate energetic compounds until the onset of metamorphosis [41] but growth rates decrease significantly once metamorphosis starts [38]. It is possible that up-regulation of some miRNAs at a pre-metamorphic/metamorphic stages is associated with changes in lipid metabolism and with the complex metamorphic process. Such alterations in lipid metabolism may also be correlated with the high expression of miR-122 during metamorphosis, as previously observed in other flatfish like halibut [19] or flounder [42]. miR-122, which is a liver-specific miRNA, has a crucial role in lipid metabolism in teleosts [43]. It is therefore plausible that changes in this miRNA abundance might be related with physiological changes occurring during metamorphosis.

Some of the miRNAs identified in Senegalese sole are known to play a stimulatory role in somatic growth and specifically in skeletal muscle development. In mammals, miR-26a up-regulation is described to post-transcriptionally repress the histone methyltransferase Ezh2, which is a suppressor of skeletal muscle cell differentiation [44]. In fish, such as the common carp, this miRNA was also associated with myogenesis [14]. In our study, the higher miR-26a expression observed in the 21°C group during hatching (and a similar trend during 75Ep and 20S) indicates a possible activation of the myogenic programme at a higher temperature. A similar conclusion can be drawn from miR-181a-5p expression in Senegalese sole embryos. miR-181 is strongly up-regulated in regenerating muscle from an *in vivo* mouse model of muscle injury [11] and endurance exercise was also found to significantly increase the expression of miR-181 in mice skeletal muscle [45]. These results are consistent with previous findings in Senegalese sole, where a lower incubation temperature (15°C) produced lighter larvae with smaller fast fibres [23].

The muscle-specific miR-206, known to reinforce the myogenic program by inhibiting the expression of DNA polymerase α (responsible for cell proliferation) and indirectly down-regulating the MyoD inhibitors Id1-3 and MyoR [46], showed increased expression levels during embryogenesis at 21°C compared to 15°C, further supporting our observations that this temperature enhanced muscle growth [23, 47]. Interestingly, MyoD was found to regulate the expression of miR-133 [48] and miR-206 [49]. Transcript levels of *myod2* at the 20S stage in Senegalese sole embryos incubated at 15°C or 21°C [23] are consistent with the expression of miR-206 observed in the present study, which is higher at 21°C than 15°C. A similar conclusion can be drawn for miR-133, based on the digital expression profiling from SOLiD sequencing. In zebrafish and medaka (*Oryzias latipes*), miR-17 is ubiquitously expressed during embryonic development but becomes restricted to proliferative tissues later in development [50]. In the present study, we observed increased expression of miR-17a-5p in the 21°C group at some developmental stages (Figure 3A); whether this is related with increased cellular proliferation is however unknown. Activation of the miR-214/199 cluster coincides with the recruitment of *myoD* and *myogenin* [51]. Furthermore, miR-199, miR-206 and miR-17 are positively associated with muscle regeneration and remodelling [52]. This ability to stimulate myogenesis may explain the differences in transcript levels of miR-199, miR-206 and miR-17 between the two temperature groups of Senegalese sole larvae at hatching. Higher levels of these miRNAs are consistent with the observed muscle phenotype in the 21°C group compared to larvae reared at 15°C [23].

The observed growth differences between temperature groups may also be associated with other metabolic functions (e.g., energy production), since the abundance of miRNAs related to lipid metabolism differed across temperature groups. For instance, the guide strand of the liver-specific miR-122 was expressed at higher levels at 15°C than at 21°C. Given the role of this miRNA in fatty-acid oxidation, and therefore in energy production, it is plausible that temperature affected lipid metabolism and energy utilisation of sole larvae, thus affecting growth. Furthermore, since the passenger strand was expressed at high levels, one can hypothesise that it may also play an active role in lipid metabolism.

### Prediction of potential targets of the differentially regulated Senegalese sole miRNAs

Several miRNAs were computationally predicted with high confidence to target mRNAs directly related to myogenesis (Figure 4, Table 2). However, it should be noted that miRNAs can have multiple mRNAs targets and whole larvae were used to investigate miRNA expression in this study. Hence, it is likely that expression levels of some Senegalese sole miRNAs reflect a broader gene regulation, except for those that are muscle-specific, such as miR-206.

mTOR and p38 MAPK pathways have overlapping yet distinct roles during myogenesis: both are required for myogenic differentiation and p38 MAPK, but not mTOR, can inhibit proliferation [6]. It was also suggested that the normoxia activation of MAPK/ERK signalling pathway not only stimulates myoblast proliferation [5] but also suppresses myogenic differentiation [6]. MAPKs can be activated by a wide variety of different stimuli, but in general MAPK1 and 3 (also called ERK2 and ERK1, respectively) are preferentially activated in response to growth factors whereas the JNK and p38 kinases (which include amongst others MAPK9 and MAPK13) are more responsive to stress stimuli like osmotic shock or ionizing radiation [53]. The major direct or indirect targets of mTOR appear to be mechanisms responsible for ribosome recruitment to mRNA, such as the eukaryotic translation initiation factor 4E (eIF4E), which is activated by the phosphorylation of its repressors, the 4EBP proteins [54, 55]. Phosphorylation of 4EBP1 by mTOR stimulates translation initiation through the release of eIF4E from 4EBP1, allowing eIF4E to associate with eIF4G to enhance cap-dependent translation [55].

The DNA-damage-inducible transcript 4 (REDD1) suppresses mTOR activity through the TSC1/TSC2 heterodimer [56, 57]. miR-181a-5p up-regulation in Senegalese sole hatchlings at 21°C may down-regulate this mTOR inhibitor. miR-181a-5p was also predicted to target the 3’ UTR of *mapk3*. Thus, the up-regulation of miR-181a-5p at 21°C during early development may have led to higher myogenic differentiation of this group. *Mapk*3 and *mapk13* are potential targets of miR-17a-5p, which are involved in MAPK/ERK and JNK/p38 MAPK signalling pathways, respectively. In human cell lines, miR-17-5p targets the 3’UTR of *Mapk9* [58]. MAPK9 is a negative regulator of cellular proliferation through the JNK/P38 MAPK pathway [59] and this interaction contributed to the proliferative phenotype caused by miR-17-5p [58]. In the present study, miR-130c was predicted to target MAPK9 mRNA and may therefore have a role in regulating cell proliferation at some developmental stages.

miR-206 was predicted to target *sestrin1,* which codes for a negative regulator of mTOR signalling through activation of AMPK and TSC2 phosphorylation [60]. The up-regulation of miR-206 at 21°C during early development may have down-regulated this myogenic inhibitor. Furthermore, *sestrin3* was predicted to be regulated by miR-17a. Like Sestrin1 and Sestrin2, Sestrin3 promotes AMPK activity [60, 61] and it has been shown that the induced expression of Sestrin3 by FoxO1 transcription factor inhibits mTOR Complex 1 (mTORC1), which regulates cell growth mostly through increased protein synthesis [61]. miR-17a up-regulation at hatching in larvae reared at 21°C is consistent with a possible down-regulation of *sestrin3* and a concomitant increase in growth.

Calpains are known to play a role in myogenesis, namely in promoting myoblast fusion [62]. Senegalese sole miR-181a-3p was predicted with high confidence to target the 3’ UTR of *calpain 1*. Its 4.9-fold up-regulation at 15°C during somitogenesis indicates a possible repression of *calpain 1*, which would impair the fusion of myoblasts to multinucleated myotubes at this temperature and result in the observed muscle phenotype differences between the two temperature groups [23].

## Conclusions

We have identified 320 conserved miRNAs in Senegalese sole, most of them dynamically expressed during early development and 48 not previously described in teleosts. Embryonic temperature affected the expression of several miRNAs, and it seems that a higher incubation temperature (21°C) promoted expression of miRNAs positively related with growth at specific developmental stages. In particular, higher expression levels of miR-17a, miR-181-5p and miR-206 and down-regulation of miR-181a-3p at 21°C may promote myogenesis and are in agreement with previous studies in Senegalese sole, which reported enhanced growth at higher embryonic temperatures compared to 15°C. Also, miRNAs related to lipid metabolism and energy production (e.g., miR-122) may also be involved in the differential growth of fish with temperature. Taken together, our data indicate that miRNAs may play a role in temperature-induced phenotypic plasticity of growth in teleosts.

## Methods

### Animal ethics

All experiments were conducted by trained scientists following FELASA category C recommendations and were carried out in accordance with the clear boundaries of EU legal frameworks, specifically those relating to the protection of animals used for scientific purposes (i.e. Directive 2010/63/EU) and under the Portuguese legislation regarding the protection of animals used for scientific purposes (Decree-Law No. 113/2013). This study was performed at the research facilities of the CCMAR (Algarve, Portugal), which has been certified for animal experimentation by the competent authority (Direcção Geral de Alimentação e Veterinária, Portugal).

### Fish husbandry

Senegalese sole embryos were incubated at two temperatures (15.2 ± 0.3°C and 20.9 ± 0.3°C) in triplicate groups at the LEOA facility, CCMAR/University of Algarve, Portugal. Eggs obtained by natural spawning of captive broodstock fish were randomly distributed amongst 6 fibreglass conical tanks (100 l) at a density of 100 eggs · l^-1^. After hatching all larvae were reared at the same temperature (20.8 ± 0.1°C) until the metamorphosis climax. Water temperature, O_2_, salinity, pH and nitrogenous compounds were monitored regularly during the entire trial, and larvae were exposed to an artificial photoperiod of 12 h:12 h light:dark. They were reared in 100 L conical tanks at a density of 40 larvae · L^-1^. At mouth opening larvae started to feed on rotifers (*Brachionus* sp.). *Artemia* nauplii were introduced at 5 days post-hatch (dph) and their density was gradually increased from 4 to 8 ml^-1^ until 8 dph. Live *Artemia* meta-nauplii were then introduced at this time point on increasing from 12 to 20 · ml^-1^ until 18 dph. Frozen meta-nauplii were offered from when larvae settled at the bottom of the tanks (around 18 dph) until 30 dph.

### Sampling and RNA extraction

Embryo and larvae samples were taken in triplicate according to the developmental stages described in Fernández-Díaz et al. [32]. The sampling points were: 75% epiboly (75Ep), 20 somites (20S), hatching (0 dph), pre-metamorphosis (Pre-Met, stage 0 according to the eye-translocation stage; 8 and 9 dph for larvae from 21°C and 15°C, respectively), metamorphosis climax (Met, stage 3 according to the eye-translocation stage; 14 dph and 15 dph in larvae incubated at 21°C and 15°C, respectively) and 30 dph. The embryos were snap-frozen in liquid nitrogen, and larvae were sacrificed by terminal anaesthesia with MS-222 (400 mg · l^-1^) and snap-frozen in liquid nitrogen. All samples were stored at -80°C until further analysis. Total RNA was extracted using the *mir*Vana miRNA isolation kit (Ambion) according to the manufacturer’s instructions. Assessment of RNA quality was performed by agarose gel electrophoresis on a 1.2% (w/v) agarose gel containing SYBR safe DNA gel stain. RNA samples were then quantified with a Nanodrop spectrophotometer (Nanodrop Technologies/Saven Werner). Absorbance ratios (260/280 nm) were greater than 1.9, indicating high purity RNA.

### SOLiD sequencing of Senegalese sole small RNAs

Small RNA reads were generated from eight SOLiD (Life Technologies, Austin, Texas, USA) libraries prepared from pools of triplicate samples of embryos and larvae from the following stages and at both 15 and 21°C temperature groups: 75Ep, 20S, hatching and metamorphosis climax stage 3. The enriched small RNA fraction of less than 40 nucleotides (nt) was recovered by sodium acetate precipitation, assessed on Agilent 2100 Bioanalyzer with small RNA chip (Agilent Technologies), and used for the amplified small RNA library template preparation using the SOLiD™ total RNA-seq kit with the barcoded SOLiD™ 3’ primers (Life Sciences). The average length of the amplified cDNA template was assessed in Agilent 2100 Bioanalyzer with the high sensitivity DNA chip kit (Agilent Technologies) to calculate the ratio of the desired miRNA ligation products (120–130 bp). To quantify the precise cDNA yield of miRNA ligation products for template bead preparation, real-time quantitative PCR (qPCR) was carried out using KAPA SYBR FAST Light Cycler 480 qPCR kit with SOLiD™ primer mix and DNA standards (Kapa Biosystems). Each library template was clonally amplified on SOLiD™ P1 beads by emulsion PCR and then sequenced on the SOLiD4 platform (Life Sciences) on a quadrant of a slide with 50 nt read length.

### Sequencing data analysis

The SOLiD raw colour space reads were pre-processed using the CLC Genomics Workbench 4.9 (CLCbio) to remove low quality reads and clip internal adaptor sequence (CTGCTGTACGGCCAAGGCC), thus leaving reads ranging between 10 and 35 nucleotides (nt) in length. Reads corresponding to small RNAs other than miRNAs (such as tRNAS, rRNA, mtRNA or snRNAs) were discarded following BLAST searches against ncRNA databases. Unannotated clusters and singletons were mapped against miRNAs sequences deposited in miRBase 18.0 (http://www.mirbase.org/) in order to identify conserved miRNAs. The remaining reads were mapped against Pleurinectiformes and Solea EST databases (NCBI) to identify possible non-conserved miRNAs. For each mappable sequence between 16 and 28 nt and with 5 counts or above, hairpin folding was evaluated by sequence analysis to identify the presence of a stem loop using the RNA secondary structure prediction tool in the CLC Genomics Workbench. The flanking EST sequence of each analysed read should be at least 60 nt, and the free energy of the fold-back structure (ΔG) should be ≤ -18 kcal · mol^-1^.

The number of reads in each sample was normalised by dividing the number of reads of a given miRNA in a sample by the total number of trimmed miRNA reads in the same sample. To be able to differentiate the expression of each miRNA and categorise them according to their expression pattern, a heatmap chart was drawn and hierarchical clustering with Euclidean distance and complete linkage algorithms was performed using the MeV online software (http://www.tm4.org/mev.html).

### Quantitative real-time PCR assay

Real-time PCR (qPCR) was used to validate the SOLiD profile of 7 selected miRNAs (mir-17a-5p, mir-26a, mir-130c, mir-206-3p, mir-181a-5p, mir-181a-3p and mir-199a-5p) using LNA™-enhanced microRNA qPCR specific primers (Exiqon A/S) (Table 3). Expression analysis of these selected miRNAs was also performed in pre-metamorphic larvae (stage 0 according to the eye-translocation stage) and in 30 dph larvae, in order to have a more complete overview of these miRNA transcript levels during Senegalese sole development.

**Table 3:** **List of primers used in quantification of miRNAs by qPCR**

MicroRNA	Mature sequence (5’ – 3’)	ID qPCR primers (Exiqon)	Amplicon (bp)	Tm (°C)	***E (%***)
**miR-26a**	UUCAAGUAAUCCAGGAUAGGCU	151790-1	49	69	98
**miR-17a-5p**	CAAAGUGCUUACAGUGCAGGUA	151777-1	45	71	90
**miR-130c**	CAGUGCAAUAUUAAAAGGGCAU	151782-1	48	69	94
**miR-206-3p**	UGGAAUGUAAGGAAGUGUGUGG	151786-1	45	70	102
**miR-199a-5p**	CCCAGUGUUCAGACUACCUGUUC	151813-1	46	70	95
**miR-181a-5p**	AACAUUCAACGCUGUCGGUGAGU	151802-1	46	71	94
**miR-181a-3p**	ACCAUCGACCGUUGAUUGUACC	151808-1	41	71	91

Amplicon sizes, melting temperatures (Tm) and PCR efficiencies (E) are indicated.

cDNA was synthesised from RNA diluted to 5 ng · μl^-1^ using the miRCURY LNA™ Universal RT microRNA PCR Kit (Exiqon) and following the manufacturer’s instructions. Reactions were performed using SYBR Green chemistry (Exiqon) on a Light Cycler 480® (Roche Applied Science) using white 96-well plates and the following running conditions: denaturation at 95°C: 10 min followed by 45 amplification cycles at 95°C: 10 s and 60°C: 1 min. Specificity of reaction was analysed by melting curve analysis [63]. Five-point standard curves of a 5-fold dilution series (1:1–1:625) of pooled RNA were used for PCR efficiency calculation [64]. All samples were run in duplicate. C_T_ values were determined using the fit-point method using the LightCycler® 480 software with a fluorescence threshold arbitrarily set to 0.3. The provided control RNA Spike-in (Exiqon) was used as exogenous reference to examine the miRNAs expression profile during development and across temperatures, using the ^ΔΔ^CT method, according to Fernandes et al. [64]. Differences in the expression of the target miRNAs between temperatures were evaluated by Student’s t-test using the SigmaPlot 11.0 statistical software (Systat Software, San Jose, CA, USA), since the normality assumption was met.

### Target prediction analysis

3’-UTRs for 3,946 annotated ESTs representing 3,397 Senegalese sole genes were obtained from NCBI-SRA and dbEST. Raw data from Senegalese sole 454 sequencing have been submitted to SRA under the experiment ID SRX246914. MiRNA target gene prediction was performed using miRanda v3.3a [65] and the RNAhybrid web server (bibiserv.techfak.uni-bielefeld.de/rnahybrid) [66]. MiRanda implements a dynamic search programme for maximal local complementarity alignments between the miRNA and the reference sequence and generate scores based on the position-specific empirical models [65]. The free energy of optimal strand-strand interaction was calculated using the ViennaRNA package incorporated within the program. Targets for Senegalese sole miRNAs were predicted using the following default parameters: Gap open penalty, -9.0; gap extend penalty, -4.0; score threshold, 140.0; energy threshold, 1.0 kcal · mol^-1^; scaling parameter, 4.0. Targets predicted to have an energy threshold below -20 kcal · mol^-1^ were selected for further analysis. The RNAhybrid web server was further used to calculate the minimum free energy of the stable duplex between miRNA and target site within the 3’-UTR.

## Electronic supplementary material

Additional file 1: Table S1: Sequences of mature miRNAs identified in Senegalese sole.The 48 miRNAs that had not been previously described in fish are highlighted in yellow. (PDF 60 KB)
