# Supplementary material for: Thermal plasticity of the miRNA transcriptome during Senegalese sole development
Source: BMC Genomics. 2014 Jun 25;15(1):525. doi: 10.1186/1471-2164-15-525 (PMC4097167; doi:10.1186/1471-2164-15-525)
Supplement: Supplementary file 1 — Additional file 1: Table S1: Sequences of mature miRNAs identified in Senegalese sole.The 48 miRNAs that had not been previously described in fish are highlighted in yellow. (PDF 60 KB) [file 12864_2013_6233_MOESM1_ESM.pdf]

Supplementary Table S1. Sequences of mature miRNAs identified in Senegalese sole.The 48 miRNAs that had not been previously described in fish are highlighted in yellow.

miRNAs	Sequence
**let-7a**	UGAGGUAGUAGGUUGUAUAGUU
**let-7b**	UGAGGUAGUAGGUUGUGUGGUU
**let-7c**	UGAGGUAGUAGGUUGUAUGGUU
**let-7d**	UGAGGUAGUUGGUUGUAUGGUU
**let-7e**	UGAGGUAGUAGAUUGAAUAGUU
**let-7f**	UGAGGUAGUAGAUUGUAUAGUU
**let-7g**	UGAGGUAGUAGUUUGUAUAGUU
**let-7h**	UGAGGUAGUAAGUUGUGUUGUU
**let-7i**	UGAGGUAGUAGUUUGUGCUGUU
**let-7j**	UGAGGUAGUUGUUUGUACAGUU
**miR-100**	AACCCGUAGAUCCGAACUUGUG
**miR-101a-3p**	UACAGUACUGUGAUAACUGAAG
**miR-101a-5p**	UCAGUUAUCACAGUGCUGAUGC
**miR-101b**	UACAGUACUAUGAUAACUGAAG
**miR-101b-5p**	UCAGUUAUCAUGGUACCGGUGCU
**miR-101c**	ACAGUACUGUGAUAACUGA
**miR-103a-3p**	AGCAGCAUUGUACAGGGCUAUGA
**miR-106a**	UAAAGUGCUUACAGUGCAGGU
**miR-107-3p**	AGCAGCAUUGUACAGGGCUAUCA
**miR-107b**	AGCAGCAUUGUACAGGGCUUU
**miR-107-5p**	AGCUUCUUUACAGUGUUGCCUUG
**miR-10a**	UACCCUGUAGAUCCGAAUUUGUG
**miR-10b**	UACCCUGUAGAACCGAAUUUGUG
**miR-10c**	UACCCUGUAGAUCCGGAUUUGU
**miR-10d**	UACCCUGUAGAACCGAAUGUGUG
**miR-1a-5p**	ACAUACUUCUUUAUAUGCCCAUA

**miR-1a-3p**	UGGAAUGUAAAGAAGUAUGUAU
**miR-1b**	UGGAAUGUAAAGAAGUAUGGGU
**miR-1c**	UGGAAUGUAAAGAAGUAUGUAC
**miR-122-3p**	AACGCCAUUAUCACACUAA
**miR-122-5p**	UGGAGUGUGACAAUGGUGUUUG
**miR-124-3p**	UAAGGCACGCGGUGAAUGCCAA
**miR-124b**	UUAAGGCACGCAGUGAAUGCCA
**miR-124-5p**	CGUGUUCACAGCGGACCUUGAU
**miR-1243**	AACUGGAUCAAUUAUAGGAGUG
**miR-125a**	UCCCUGAGACCCUUAACCUGUG
**miR-125b-5p**	UCCCUGAGACCCUAACUUGUGA
**miR-125c**	UCCCUGAGACCCUAACUUGUGAC
**miR-126b**	UCGUACCGUGAGUAAUAGUGCA
**miR-126a-3p**	UCGUACCGUGAGUAAUAAUGC
**miR-126a-5p**	CAUUAUUACUUUUGGUACGCG
**miR-1260a**	AUCCCACCUCUGCCACCA
**miR-1260b**	AUCCCACCACUGCCACCAU
**miR-128**	UCACAGUGAACCGGUCUCUUU
**miR-129-5p**	CUUUUUGCGGUCUGGGCUUGCU
**miR-130a**	CAGUGCAAUGUUAAAAGGGCAU
**miR-130b-3p**	CAGUGCAAUAAUGAAAGGGCAU
**miR-130b-5p**	ACUCUUUCCCUGUUGCACUACU
**miR-130c**	CAGUGCAAUAUUAAAAGGGCAU
**miR-132-3p**	UAACAGUCUACAGCCAUGGUCG
**miR-133a-3p**	UGGUCCCCUUCAACCAGC
**miR-133a-5p**	AGCUGGUAAAAUGGAACCAAAUC
**miR-133b-3p**	UUUGGUCCCCUUCAACCAGCUA
**miR-133b-5p**	GCUGGUCAAACGGAACCAAGUC
**miR-133c**	UUUGGUCCCUUUCAACCAGCUA
**miR-133d**	UUGGUCCCCUUCAACCAGCCGC

**miR-135a**	UAUGGCUUUUUAUUCCUAUGUGA
**miR-135b**	UAUGGCUUUUUAUUCCUAUCUG
**miR-135c**	UAUGGCUUUCUAUUCCUAUGUG
**miR-137a-3p**	UUAUUGCUUAAGAAUACGCGUAG
**mir-137b**	UUAUUGCUUGAGAAUACGCGUA
**miR-137a-5p**	ACGGGUAUUCUUGGGUGGAUAAU
**miR-138a**	AGCUGGUGUUGUGAAUCAGGCCG
**miR-138b**	AGCUGGUGUUGUGAAUCAGGCUG
**miR-1388-3p**	AUCUCAGGUUCGUCAGCCCAUG
**miR-1388-5p**	AGGACUGUCCAACCUGAGAAUG
**miR-139**	UCUACAGUGCAUGUGUCUCCA
**miR-140-5p**	CAGUGGUUUUACCCUAUGGUAG
**miR-141**	UAACACUGUCUGGUAACGAUGC
**miR-142**	CCCAUAAAGUAGAAAGCACUAC
**miR-142b**	CAUAAAGUAGAAAGCACUACUA
**miR-143-3p**	UGAGAUGAAGCACUGUAGCUC
**miR-143-5p**	GGUGCAGUGCUGCAUCUCUGGU
**miR-144-3p**	UACAGUAUAGAUGAUGUACU
**miR-144-5p**	AGGAUAUCAUCUUAUACUGUAA
**miR-145**	GUCCAGUUUUCCCAGGAAUCCCU
**miR-146a**	UGAGAACUGAAUUCCAUAGAUGG
**miR-146b**	UGAGAACUGAAUUCCAUAGGCU
**miR-147b**	GUGUGCGGAAAUGCUUCUGC
**miR-148**	UCAGUGCAUUACAGAACUUU
**miR-150**	ACUCCCAAUCCUUGUACCAGU
**miR-152-3p**	UCAGUGCAUGACAGAACUUUGG
**mir-152b**	UCAGUGCAAAACAGAACUUUG
**mir-152-5p**	UAGGUUCUGUGAUACACUCCGACU
**miR-153a**	UUGCAUAGUCACAAAAGUGAUC
**miR-153b**	UUGCAUAGUCACAAAAAUGAGC

**miR-153c**	UUGCAUAGUCACAAAAAUGAUC
**miR-155**	UUAAUGCUAAUCGUGAUAGGGGU
**miR-15a**	UAGCAGCACGGAAUGGUUUGUG
**miR-15b**	UAGCAGCGCAUCAUGGUUUGUA
**miR-1582**	GAAAGAGAGCCAGAACACAG
**miR-1599**	GGAGGGAGGAAAAAAAAAAA
**miR-16a**	UAGCAGCACGUAAAUAUUGGUG
**miR-16b**	UAGCAGCACGUAAAUAUUGGAG
**miR-16c**	UAGCAGCAUGUAAAUAUUGGAG
**miR-1623**	GCAGGCACAGACAGGCAGUA
**miR-1692**	UGUAGCUCAGUUGGUAGAGU
**miR-17a-5p**	CAAAGUGCUUACAGUGCAGGUA
**miR-1788-5p**	GGCUUGUUUUAAGUUGCCUGCG
**miR-1788-3p**	CAGGCAGCUAAAGCAAGUC
**miR-1701**	GGCUGGUUAGUUGGUUGUUU
**miR-18a**	UAAGGUGCAUCUAGUGCAGAUA
**miR-18b**	UAAGGUGCAUCUAGUGCAGUUAG
**miR-18c**	UAAGGUGCAUCUUGUGUAGUUA
**miR-181a-5p**	AACAUUCAACGCUGUCGGUGAGU
**miR-181a-3p**	ACCAUCGACCGUUGAUUGUACC
**miR-181b**	AACAUUCAUUGCUGUCGGUGGGUU
**miR-181c**	CACAUUCAUUGCUGUCGGUGGG
**miR-181d**	AACAUUCAUUGUUGUCGGUGGGU
**miR-182-5p**	UUUGGCAAUGGUAGAACUCACA
**miR-182-3p**	UGGUUCUAGACUUGCCAACUA
**miR-183**	UAUGGCACUGGUAGAAUUCACUG
**miR-184-3p**	UGGACGGAGAACUGAUAAGGGC
**miR-184-5p**	UCCUUAUCACUUUUCCAGCCCA
**miR-187-3p**	UCGUGUCUUGUGUUGCAGCC
**miR-187-5p**	GGCUGCAACACAGGACAU

**miR-188**	CAUCCCUUGCAUGGUGGAGGG
**miR-190a**	UGAUAUGUUUGAUAUAUUAGGU
**miR-190b**	UGAUAUGUUUGAUAUUCGGUUG
**miR-192**	AUGACCUAUGAAUUGACAGCC
**miR-193a**	AACUGGCCUACAAAGUCCCAGU
**miR-194a**	UGUAACAGCAACUCCAUGUGG
**miR-194b**	UGUAACAGCCGCUCCAUGUGGA
**miR-1957a**	CAGUGGUAGAGCAUAUGAC
**miR-1973**	ACCGUGCAAAGGUAGCAUA
**miR-196a**	UAGGUAGUUUCAUGUUGUUGGG
**miR-196b**	UAGGUAGUUUCAAGUUGUUGGG
**miR-196c**	UAGGUAGUUUGAUGUUGUUGGG
**miR-196d**	UAGGUAGUUUUAUGUUGUUGGG
**miR-199a-5p**	CCCAGUGUUCAGACUACCUGUUC
**miR-199a-3p**	CAGUAGUCUGCACAUUGGUUA
**miR-199b**	CCCAGUGUUUAGACUACCUGUUC
**miR-19a-3p**	UGUGCAAAUCUAUGCAAAACUGA
**miR-19a-5p**	CUAGUUUUGCAUAGUUGCACUA
**miR-19b-3p**	UGUGCAAAUCCAUGCAAAACUGA
**miR-19c**	UGUGCAAAUCCAUGCAAAACUCG
**miR-19d**	UGUGCAAACCCAUGCAAAACUGA
**miR-20a-5p**	UAAAGUGCUUAUAGUGCAGGUAG
**miR-20a-3p**	ACUGCAGUGUGAGCACUUGAAG
**miR-20b**	CAAAGUGCUCACAGUGCAGGUAG
**miR-200a-3p**	UAACACUGUCUGGUAACGAUGU
**miR-200a-5p**	CAUCUUACCGGACAGUGCUGGA
**miR-200b-3p**	UAAUACUGCCUGGUAAUGAUGAU
**miR-200b-5p**	CAUCUUACUGGGCAGCAUUGGA
**miR-200c**	UAAUACUGCCUGGUAAUGAUGC
**miR-202-5p**	UUCCUAUGCAUAUACCUCUUUG

**miR-202-3p**	AGAGGCAUAGGGCAUGGGAAAA
**miR-203a**	GUGAAAUGUUUAGGACCACUUG
**miR-203b-3p**	GUGAAAUGUUCAGGACCACUUG
**miR-203b-5p**	AGUGGUUCUUGACAGUUCAACA
**miR-204**	UUCCCUUUGUCAUCCUAUGCCU
**miR-204b**	UUCCCUUUGUUAUCCUAUGCCU
**miR-205-5p**	UCCUUCAUUCCACCGGAGUCUG
**miR-205b**	CCCUUCAUUCCACCGGAGUCUGU
**miR-205-3p**	AGAUUUCAGUGGUGUGAAGUGU
**miR-206-3p**	UGGAAUGUAAGGAAGUGUGUGG
**miR-206-5p**	ACAUGCUUCUUUAUAUCCUCAUA
**miR-21**	UAGCUUAUCAGACUGGUGUUG
**miR-210-3p**	CUGUGCGUGUGACAGCGGCUAA
**miR-210-5p**	AGCCACUGACUAACGCACAU
**miR-211**	UUCCCUUUGUCAUCCUUCGCCU
**miR-212-3p**	UAACAGUCUACAGUCAUGGCU
**miR-212-5p**	ACCUUGGCUCUAGACUGCUUACU
**miR-2137**	GCCGGCGGGAGCCCCAGGGAG
**miR-214-3p**	ACAGCAGGCACAGACAGGCAG
**miR-214-5p**	UGCCUGUCUACACUUGCUGUGC
**miR-215**	AUGACCUAUGAAUUGACAGAC
**miR-216b**	UAAUCUCUGCAGGCAACUGUGA
**miR-216a**	AAAUCUCAGCUGGCAACUGUGA
**miR-217**	UACUGCAUCAGGAACUGAUUGG
**miR-218a**	UUGUGCUUGAUCUAACCAUGUG
**miR-218b**	UUGUGCUUGAUCUAACCAUGCA
**miR-2184**	AACAGUAAGAGUUUAUGUGCU
**miR-2187-3p**	UUACAGGCUAUGCUAAUCUAUG
**miR-2188**	AAGGUCCAACCUCACAUGUCCU
**miR-219**	UGAUUGUCCAAACGCAAUUCUU

**miR-22a**	AAGCUGCCAGCUGAAGAACUGU
**miR-22b**	AAGCUGCCAGUUGAAGAGCUGU
**miR-22-3p**	AAGCUGCCAGUUGAAGAACUGU
**miR-221-3p**	AGCUACAUUGUCUGCUGGGUUUC
**miR-221-5p**	ACCUGGCAUACAAUGUAGAUUUCUGU
**miR-222a-3p**	AGCUACAUCUGGCUACUGGGUCUC
**miR-222-5p**	CUCAGUAGCCAGUGUAGAUCCU
**miR-223-3p**	UGUCAGUUUGUCAAAUACCCC
**miR-223-5p**	CGUGUAUUUGACAAGCUGAGUU
**miR-23a-3p**	AUCACAUUGCCAGGGAUUUCCA
**miR-23b**	AUCACAUUGCCAGGGAUUACCAC
**miR-23c**	AUCACAUUGCCAGUGAUUACCC
**miR-24a-3p**	UGGCUCAGUUCAGCAGGAACAG
**miR-24a-5p**	GUGCCUACUGAACUGGUAUCAGU
**miR-24b**	UGGCUCAGUUCAGCAGGACAG
**miR-24c**	UGGCUCAGUUCAGCAGAA
**miR-25-3p**	CAUUGCACUUGUCUCGGUCUGA
**miR-25-5p**	AGGCGGAGACUUGGGCAAUUG
**miR-252**	AUAAGUAGUAGUGCCGCAGGUAA
**miR-258**	GGUUUUGAGAGGAAUCCUUUU
**miR-26a**	UUCAAGUAAUCCAGGAUAGGCU
**miR-26b**	UUCAAGUAAUCCAGGAUAGGUU
**miR-27a**	UUCACAGUGGCUAAGUUCCG
**miR-27b-3p**	UUCACAGUGGCUAAGUUCUGCA
**miR-27b-5p**	AGAGCUUAGCUGAUUGGUGAAC
**miR-27c**	UUCACAGUGGUUAAGUUCUGC
**miR-27d**	UUCACAGUGGUUAAGUUCUGC
**miR-27e**	UUCACAGUGGCUAAGUUCAGUG
**miR-281a**	UGUCAUGGAGUUGCUCUCUUGC
**miR-29a**	UAGCACCAUUUGAAAUCGGUUA

**miR-29b**	UAGCACCAUUUGAAAUCAGUGU
**miR-29d**	UAGCACCAUAUGAAAUCGGUGUC
**miR-301a-3p**	CAGUGCAAUAGUAUUGUCAAAG
**miR-301a-5p**	GCUCUGACUUCAUUGCACUAC
**miR-301b**	CAGUGCAAUAGUAUUGUCAUUG
**miR-301b-5p**	GCUCUGACAAUGUUGCACUAC
**miR-301c**	CAGUGCAAUAGUAUUGUCAUAG
**miR-30a-5p**	UGUAAACAUCCUCGACUGGAAG
**miR-30a-3p**	CUUUCAGUCGGAUGUUUGCAGC
**miR-30b**	UGUAAACAUCCUACACUCAGCU
**miR-30c**	UGUAAACAUCCUACACUCUCAGCU
**miR-30d**	UGUAAACAUCCCCGACUGGAAG
**miR-30e-5p**	UGUAAACAUCCUUGACUGG
**miR-30f**	UGUAAACACCCUACACUCUCAGC
**miR-31**	AGGCAAGAUGUUGGCAUAGCUG
**mirR-320a**	AAAAGCUGGGUUGAGAGGGCGA
**mirR-320d**	AAAAGCUGGGUUGAGAGGA
**miR-33-3p**	CAAUGUACCUGCAGUGCAA
**miR-33-5p**	GUGCAUUGUAGUUGCAUUGCA
**miR-338-3p**	UCCAGCAUCAGUGAUUUUGUUG
**miR-338-5p**	AACAACAUCCUGGUGCUGCCUGAGU
**miR-34a**	UGGCAGUGUCUUAGCUGGUUGU
**miR-365**	UAAUGCCCCUAAAAAUCCUUAU
**miR-363**	AAUUGCACGGUAUCCAUCUGUA
**miR-3618**	UGUCUACAUUAAUGAAAAGAGC
**miR-375**	UUUGUUCGUUCGGCUCGCGUUA
**miR-3963**	UGUAUCCCACUUCUGACAC
**miR-3968**	CGAAUCCCACUCCAGACACCA
**miR-3975**	UGAGGCUAAUGCACUACUUCAC
**miR-4286**	ACCCCACUCCUGGUACC

**miR-4255**	CAGUGUUCAGAGAUGGA
**miR-4256**	AUCUGACCUGAUGAAGGU
**miR-4273**	GUGUUCUCUGAUGGACAG
**miR-4289**	GCAUUGUGCAGGGCUAUCA
**miR-4295**	CAGUGCAAUGUUUUCCUU
**miR-429-3p**	UAAUACUGUCUGGUAAUGCCGU
**miR-429-5p**	GUCUUACCAGACAUGGUUAGA
**miR-430a**	UAAGUGCUAUUUGUUGGGGUAG
**miR-430d**	UAAGUGCUUCUCUUUGGGGUUG
**miR-430c**	UAAGUGCUUCUCUUUGGGGUAG
**miR-430e**	UAAGUGCUAUGUUGUUGGGGAAU
**miR-430i**	UAAGUGCUAUUUGUUGGCGUAG
**miR-4304**	CCGGCAUGUCCAGGGCA
**miR-4319**	UCCCUGAGCAAAGCCAC
**miR-4327**	GGCUUGCAUGGGGGACUGG
**miR-449b**	AGGCAGUGUAUUGUUAGCUGGC
**miR-4448**	GGCUCCUUGGUCUAGGGGUA
**miR-4454**	GGAUCCGAGUCACGGCACCA
**miR-4502**	GCUGAUGAUGAUGGUGCUGAAG
**miR-454**	UAGUGCAAUAUUGCUUAUAGGGU
**miR-456**	CAGGCUGGUUAGAUGGUUGUCA
**miR-458**	AUAGCUCUUUAAAUGGUACUGC
**miR-459**	UCAGUAACAAGGAUUCAUCCUG
**miR-4500**	UGAGGUAGUAGUUUCUU
**miR-4532**	CCCCGGGGAGCCCGGCG
**miR-4576**	UUUGUUUAGCGAUGUAUCUGGC
**miR-451**	AAACCGUUACCAUUACUGAGUU
**miR-455a**	UAUGUGCCCUUGGACUACAUCG
**miR-455b**	GUAUGUGCCCUUGGACUACAUU
**miR-4560**	UCUCUGUCCACACAAACACCUG

**miR-460-5p**	CCUGCAUUGUACACACUGUGCG
**miR-460-3p**	CACAGCGCAUACAAUGUGGAUG
**miR-460b**	UCCUCAUUGUACAUGCUGUGUG
**miR-462**	UAACGGAACCCAUAAUGCAGCU
**miR-466i**	UGUGUGUGUGUGUGUGUGUG
**miR-466q**	GUGCACACACACACAUACGU
**miR-467g**	UAUACAUACACACACAUAUAU
**miR-467f**	AUAUACACACACACACCUACA
**miR-4792**	CGGUGAGCGCUCGCUGGC
**miR-489**	AGUGACAUCAUAUGUACGGCUGC
**miR-499-5p**	UUAAGACUUGCAGUGAUGUUUA
**miR-499-3p**	AACAUCACUUUAAGUCUGUGC
**miR-509**	UACUGCAGACAGUGGCAAUCA
**miR-5100**	UCGAAUCCCAGCGGUGCCUCU
**miR-5108**	GUAGAGCACUGGAUGGUUU
**miR-5112**	UAGCUCAGUGGUAGAG
**miR-5119**	CAUCUCAUCCUGGGGCUGG
**miR-5124a**	GGUCCAGUGACUAAGAGCAU
**miR-574**	UGAGUGUGUGUGUGUGAGUGUGU
**miR-7a**	UGGAAGACUAGUGAUUUUGUUGUG
**miR-7b**	UGGAAGACUAGUGAUUUUUGUU
**miR-71**	UGAAAGACAUGGGUAGUGAGACG
**miR-72**	AGGCAAGAUGUUGGCAUAGCUGA
**miR-722**	UUUUUUGCAGAAACGUUUCAGAUU
**miR-724**	UUAAAGGGAAUUUGCGACUGUU
**miR-725**	UUCAGUCAUUGUUUCUAGUAGU
**miR-727-3p**	GUUGAGGCGAGUUGAAGACUUA
**miR-727-5p**	UCAGUCUUCAAUUCCUCCCAGC
**miR-728**	AUACUAAGUACACUACGUUUUC
**miR-730**	UCCUCAUUGUGCAUGCUGUGUGU

**miR-731**	AAUGACACGUUUUCUCCCGGAUCG
**miR-733**	UGCGUUGGUUUAGCUCAGUGGUU
**miR-737**	AAUCAAAACCUAAAGAAAAUA
**miR-9a-3p**	AUAAAGCUAGAUAACCGAAAG
**miR-9a-5p**	UCUUUGGUUAUCUAGCUGUAUGA
**miR-92a**	UAUUGCACUUGUCCCGGCCUGU
**miR-92b**	UAUUGCACUUGUCCCGGCCUCCC
**miR-93**	AAAAGUGCUGUUUGUGCAGGUA
**miR-96**	UUUGGCACUAGCACAUUUUUGCU
**miR-99a-5p**	AACCCGUAGAUCCGAUCUUGUG
**miR-99a-3p**	CAAGCUCGCCUCUGUGGGUCUC
**miR-99b**	CACCCGUAGAACCGACCUUGCG
**miR-140-3p**	UACCACAGGGUAGAACCACGGAC
**miR-129-3p**	AAGCCCUUACCCCAAAAAGCAU
**miR-9b-3p**	UAAAGCUAGAGAACCGAAUGU
**miR-458a-5p**	AGCGCCAUUUUCAGAGCUAUA
**miR-132-5p**	ACCGUGGCAUUAGAUUGUUACU
**miR-2188-3p**	CUGUGUGAGGUUAGACCUAUC
**miR-17a-3p**	ACUGCAGUGGAGGCACUUCUAG
**miR-23a-5p**	UGGGGUUCCUGGCACGGUGAUUU
**miR-2187-5p**	UUAAUUAGUAUAGCCUGUUUUA
**miR-19b-5p**	AGUUUUGCUGGUUUGCAUUCAG
**miR-130c-5p**	GCCCUUUUUAUGUUGUACUACU
**miR-125b-3p**	ACUGGUUAGGCUCUUGGGAAC
